# Population dataset for 21 simple tandem repeat loci in the Akan population of Ghana

**DOI:** 10.1016/j.dib.2020.105746

**Published:** 2020-05-22

**Authors:** Abban Edward Kofi, Hashom Mohd Hakim, Hussein Omar Khan, Siti Afifah Ismail, Anita Ghansah, Abd Rashid Nur Haslindawaty, Shaharum Shamsuddin, Mohd Yusmaidie Aziz, Geoffrey Keith Chambers, Hisham Atan Edinur

**Affiliations:** a School of Health Sciences, Universiti Sains Malaysia, Health Campus, 16150 Kubang Kerian, Kelantan, Malaysia; b Forensic Science Laboratory, Criminal Investigation Department, Ghana Police Service 233 Accra Ghana; c DNA Databank Division (D13), Criminal Investigation Department, Royal Malaysia Police, 50560, Bukit Aman, Kuala Lumpur, Malaysia; d Nugochi Memorial Institute of Medical Research, University of Ghana, 233 Accra, Ghana; e Integrative Medicine Cluster, Advanced Medical and Dental Institute, Universiti Sains Malaysia, 13200 Bertam, Kepala Batas, Penang, Malaysia; f School of Biological Sciences, Victoria University of Wellington, P.O. Box 600, Wellington, 6140, New Zealand; g Institute of Tropical Biodiversity and Sustainable Development, Universiti Malaysia Terengganu, 21030 Kuala Nerus, Terengganu, Malaysia; h Environmental Futures Research Institute, Griffith University, Nathan, Queensland 4111, Australia

**Keywords:** STR, Investigator 24plex, Akan, Ghana

## Abstract

Short tandem repeat (STR) loci are widely used as genetic marker for ancestral and forensic analyses. The latter application includes for paternity testing and DNA profiling of samples collected from scenes of crime and suspects. This survey provides the first dataset for 21 STR loci across the Akan population in Ghana by genotyping of 109 unrelated healthy individuals using Investigator 24plex kit. None of the STR loci screened deviated from Hardy-Weinberg equilibrium after applying Bonferroni correction. Overall, 224 unique alleles were observed with allele frequencies ranging from 0.005 to 0.518. The combined match probability, combined power of exclusion and combined power discrimination were 1 in 4.07 × 10^−25^, 0.999999999 and 1, respectively. Principal coordinate analysis carried out using 21 STR allele frequency data mapped the Akans with Nigerian subpopulation groups (Hausa, Igbo and Yoruba), but separated from Thais of Thailand, Chechen of Jordan and Tijuana of Mexico.

**Specifications Table**

**Table:** 

**Subject**	Genetics
**Specific subject area**	DNA profiling
**Type of data**	Tables and figure
**How data were acquired**	Capillary electrophoresis of STR polymerase chain reaction amplified products on 3500XL Genetic Analyser (Applied Biosystems, USA)
**Data format**	Raw and analyzed
**Parameters for data collection**	Genomic DNA samples extracted from cheek cells were used as templates for amplification of 21 STR loci using Investigator 24plex QS kits (Qiagen, Germany)
**Description of data collection**	Comparison of separated STR fragments with standard allelic ladder included in the Investigator 24plex QS kit using the GeneMapper IDx v4.1 software (Applied Biosystems, USA).
**Data source location**	Forensic Science Program, School of Health Sciences, Health Campus, 16150 Kubang Kerian, Kelantan, Malaysia
**Data accessibility**	Data available in this article

**Value of the data**

• In the Sub-Saharan region of Africa, population data for these 21 STR loci are only available for Nigerian subpopulations. The 21 STR dataset for the Akans of Ghana reported in this article is thus the first from a different country.
• Our 21 locus STR dataset provides an important source of information to estimate their statistical value as DNA evidence (match probability, power of exclusion power etc.) for this population group
• Data from this survey also supports the general value of STR loci for population studies across the region; allele frequencies of the 21 STR loci can be used to examine the past history of population events in Akans including gene flow, natural selection and migration patterns.
• The population genetic data and forensic statistics reported for the Akans can be used as a reference standard in future studies of other sub-population groups in Ghana; Ewe, Mole-Dagbon, Ga-Dangbe and Guang.

## 1 Data description

The allelic scores for the 21 STR loci examined across 109 unrelated Akan individuals are shown in Supplementary Table 1. Their allele frequency data and forensic parameters are shown in Table 1. A total of 224 unique alleles were observed with corresponding allele frequency ranging from 0.005 to 0.518. The observed heterozygosity (Ho) ranged from 0.725 (THO1, DS5818 and D7S820 loci) to 0.917 (SE33 locus) while the expected heterozygosity (He) ranged from 0.669 (TH01 locus) to 0.927 (SEE33 locus). After applying Bonferroni correction (*p* = 0.05/21, at 95% significance level), no deviation from Hardy-Weinberg equilibrium (HWE)were observed. The highest power of discrimination (PD) and polymorphic information content (PIC) were 0.982 and 0.917 (respectively), recorded for the SE33 locus. Locus TH01 showed the lowest PIC (0.628) and PD (0.824) values. The combined matching probability (CMP), combined probability of exclusion (CPE) and combined discriminating power (CPD) were 1 in 4.07 × 10^−25^, 0.999999999 and 1, respectively.

**Table 1:** Allele frequency data and forensic parameters for Akans of Ghana (n=109)

Allele	TH01	D3S1358	VWA	D21S11	TPOX	D1S1656	D12S391	SE33	D10S1248	D22S1045	D19S433	D8S1179	D2S1338	D2S441	D18S51	FGA	D16S539	CSF1PO	D13S317	D5S818	D7S820
5	0.009																				
6	0.087				0.087																
7	0.518				0.023													0.096			0.009
8	0.211				0.271				0.005	0.009							0.014	0.078	0.023	0.055	0.220
9	0.101				0.257						0.005	0.009					0.220	0.028	0.009	0.005	0.119
9.3	0.055																				
10	0.018				0.110	0.014			0.005	0.032	0.009	0.009		0.023			0.124	0.252	0.009	0.060	0.358
11					0.220	0.037			0.050	0.096	0.110	0.028		0.362	0.009		0.358	0.239	0.252	0.234	0.239
11.3														0.128							
12		0.005			0.023	0.069		0.005	0.138	0.064	0.101	0.133		0.124	0.083		0.138	0.239	0.472	0.353	0.055
12.2											0.041										
13			0.028		0.005	0.133		0.014	0.174		0.303	0.147		0.106	0.009		0.147		0.174	0.257	
13.2											0.050										
14		0.064	0.050		0.005	0.239	0.005	0.046	0.312	0.174	0.179	0.339		0.225	0.041			0.069	0.050	0.032	
14.2											0.064										
15		0.298	0.280			0.188	0.087	0.050	0.220	0.197	0.037	0.257		0.032	0.211				0.009		
15.2											0.069										
15.3						0.023															
16		0.427	0.220			0.115	0.069	0.050	0.078	0.179		0.046	0.064		0.156					0.005	
16.2											0.032										
16.3						0.087															
17		0.161	0.220			0.028	0.133	0.092	0.018	0.206		0.028	0.073		0.170	0.005					
17.3						0.023															
18		0.041	0.110			0.005	0.257	0.124		0.037		0.005	0.023		0.115	0.028					
18.2																0.009					
18.3						0.032	0.005														
19		0.005	0.055			0.009	0.183	0.138		0.005			0.151		0.106	0.055					
19.2																0.014					
20			0.032				0.138	0.096					0.050		0.060	0.018					
20.2																0.014					
21			0.005				0.050	0.060					0.174		0.023	0.083					
21.2																0.009					
22							0.050	0.028					0.133		0.005	0.206					
22.2								0.009								0.009					
23							0.005						0.101		0.009	0.142					
23.2								0.018								0.014					
24				0.005			0.018						0.119		0.005	0.165					
24.2								0.018								0.005					
25													0.087			0.087					
25.2								0.055								0.005					
26				0.005									0.014			0.073					
26.2								0.060													
27				0.018									0.009			0.037					
27.2								0.069													
28				0.303												0.018					
28.2								0.032													
29				0.202												0.005					
29.2								0.023													
30				0.165																	
30.2				0.014				0.014													
31				0.083																	
31.2				0.028																	
32				0.028																	
32.2				0.073																	
33				0.014																	
33.2				0.014																	
34				0.009																	
35				0.037																	
36				0.005																	
N	7	7	9	16	9	14	12	20	9	10	12	10	12	7	14	21	6	7	8	8	6
MP	0.176	0.149	0.066	0.052	0.089	0.039	0.050	0.018	0.091	0.052	0.055	0.087	0.029	0.099	0.050	0.033	0.093	0.082	0.149	0.109	0.112
PD	0.824	0.851	0.934	0.948	0.911	0.961	0.950	0.982	0.909	0.948	0.945	0.913	0.971	0.901	0.950	0.967	0.907	0.918	0.851	0.891	0.888
PIC	0.628	0.648	0.778	0.804	0.760	0.846	0.828	0.917	0.767	0.820	0.822	0.744	0.873	0.743	0.851	0.873	0.733	0.773	0.631	0.706	0.709
PE	0.468	0.514	0.530	0.530	0.665	0.665	0.756	0.831	0.630	0.630	0.701	0.530	0.701	0.665	0.812	0.756	0.579	0.683	0.370	0.468	0.468
TPI	1.817	2.019	2.096	2.096	3.028	3.028	4.192	6.056	2.725	2.725	3.406	2.096	3.406	3.028	5.450	4.192	2.370	3.206	1.473	1.817	1.817
Ho	0.725	0.752	0.761	0.761	0.835	0.835	0.881	0.917	0.817	0.817	0.853	0.761	0.853	0.835	0.908	0.881	0.789	0.844	0.661	0.725	0.725
He	0.669	0.701	0.809	0.828	0.795	0.864	0.850	0.927	0.800	0.844	0.842	0.779	0.888	0.777	0.869	0.887	0.771	0.805	0.683	0.750	0.753
*p*-HWE	0.008	0.876	0.252	0.756	0.634	0.296	0.596	0.818	0.127	0.470	0.446	0.183	0.774	0.006	0.034	0.162	0.427	0.348	0.521	0.249	0.036

N, number of loci; MP, matching probability; PD, power of discrimination; PIC, polymorphic information content; PE, power of exclusion; TPI, typical paternity index; Ho, observed heterozygosity; He, expected heterozygosity; *p*-HWE, p-value for Hardy-Weinberg equilibrium.

**Fig. 1:**
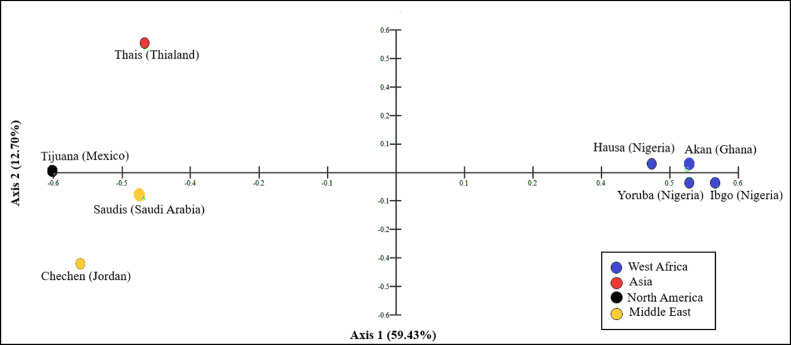
PCO plot of 21 STR loci allele frequency data obtained from present survey of Akans and other reference populations.

Principal coordinate (PCO) mapping performed using the allele frequency data across all 21 STR loci in the Akans and 7 other previously reported STR datasets (Supplementary Table 2) is shown in Fig. 1. The first and second axes accounted for 59.43% and 12.71% of genetic variability between datasets. The populations from Asia and North America are clustered in the upper left-hand quadrant. In contrast, the populations of Middle Eastern origin are plotted on the lower left quadrant. The Akans plotted closely with the Nigerian subpopulations in the lower right quadrant.

## 2 Experimental design, materials, and methods

### 2.1 Ethical clearance

This research was reviewed and approved by the Institutional Review Board of Nugochi Memorial Institute of Medical Research (NMIMR), University of Ghana (permit no: NMIMR-IRB CPN 118/15-16 *revd.* 2019) and the Human Ethics Committee of University Sains Malaysia (USMKK), Health Campus, Kelantan, Malaysia (permit no: USM/JEPeM/16050188).

### 2.2 Sample collection

A total of 109 healthy unrelated individuals of Akan ethnicity aged between 18 to 45 years were recruited for this research. Each individual provided written informed consent and have at least three generations of un-admixed history. The sampling locations included Accra, Koforidua and Kumasi of Ghana.

### 2.3 DNA isolation and STR amplification

Cheek cells were collected using buccal swab sticks and genomic DNA was extracted from them using Invisorb® Spin Forensic kit (STRATEC Molecular GmbH, Germany). Total genomic DNA was quantified using Investigator Quantiplex Hyres kit according to manufacturer's recommendation (Qiagen, Hilden, Germany). The Investigator 24plex QS amplification kits (Qiagen, Germany) was utilized to amplify the sex-determining marker Amelogenin, 1 Y-STR locus, 2 quality sensors and 21 autosomal STR loci namely CSF1PO, D10S1248, D12S391, D13S317, D16S539, D18S51, TPOX, D19S433, D1S1656, D21S11, D22S1045, D2S1338, D2S441, D3S1358, D5S818, D7S820, FGA, D8S1179, SE33, TH01 and vWA on the GeneAmp PCR System 9700 thermal cycler (Applied Biosytems, USA) in total of 12.5 ul reaction volume. PCR products were separated by multi-capillary electrophoresis in 3500XL Genetic Analyzer (Applied Biosystems, Foster City, CA, USA). The STR alleles were systematically called using the GeneMapper IDx v4.1 software (Applied Biosystems, USA). Allele designations were determined by comparison of the sample fragments with those of allelic ladders provided in the kit.

### 2.4 Statistical analysis

The HWE and the expected heterozygosity (He) values were calculated using Arlequin v3.5.2.2 [1,2]. The significance level for deviation from HWE (<0.05) was adjusted to *p* > 0.00238 after Bonferroni correction (*p* = 0.05/21 = 0.00238, where 21 is the number of loci and 0.05 is the standard HWE significance value). Allele frequencies for the 21 STR loci, PD, power of exclusion (PE), MP, typical paternity index (TPI), PIC and Ho values were computed using the Powerstats software version 1.2 [3]. PCO data mapping was used to compare and visualize genetic relatedness between Akans (Ghana), Thais [4], Tijuanans [5], Chechens living in Jordan [6], Saudis [7], and Nigerians [8]). The PCO analysis was performed using the MVSP software version 3.22 [9] and STR datasets for PCO analysis are provided as Supplementary table 2.

## Declaration of Competing Interest

The authors declare that they have no known competing interests or personal relationships that could have appeared to influence the work reported in this paper.

## Appendix Supplementary materials

Image, application 1

Image, application 2

Image, application 3
